# Unravelling the relationship between amyloid accumulation and brain network function in normal aging and very mild cognitive decline: a longitudinal analysis

**DOI:** 10.1093/braincomms/fcac282

**Published:** 2022-11-02

**Authors:** Gemma Moffat, Peter Zhukovsky, Gillian Coughlan, Aristotle N Voineskos

**Affiliations:** Kimel Family Translational Imaging-Genetics Laboratory, Campbell Family Mental Health Research Institute, Centre for Addiction and Mental Health, Toronto, ON M5T 1R8, Canada; Kimel Family Translational Imaging-Genetics Laboratory, Campbell Family Mental Health Research Institute, Centre for Addiction and Mental Health, Toronto, ON M5T 1R8, Canada; Department of Psychiatry, University of Toronto, Toronto, ON M5T 1R8, Canada; Rotman Research Institute, Baycrest Hospital, Toronto, ON, M6A 2E1, Canada; Department of Neurology, Massachusetts General Hospital, Boston, MA 02129, USA; Kimel Family Translational Imaging-Genetics Laboratory, Campbell Family Mental Health Research Institute, Centre for Addiction and Mental Health, Toronto, ON M5T 1R8, Canada; Department of Psychiatry, University of Toronto, Toronto, ON M5T 1R8, Canada

**Keywords:** amyloid, preclinical, Alzheimer’s, resting-state fMRI, functional connectivity

## Abstract

Pathological changes in the brain begin accumulating decades before the appearance of cognitive symptoms in Alzheimer’s disease. The deposition of amyloid beta proteins and other neurotoxic changes occur, leading to disruption in functional connections between brain networks. Discrete characterization of the changes that take place in preclinical Alzheimer’s disease has the potential to help treatment development by targeting the neuropathological mechanisms to prevent cognitive decline and dementia from occurring entirely. Previous research has focused on the cross-sectional differences in the brains of patients with mild cognitive impairment or Alzheimer’s disease and healthy controls or has concentrated on the stages immediately preceding cognitive symptoms. The present study emphasizes the early preclinical phases of neurodegeneration. We use a longitudinal approach to examine the brain changes that take place during the early stages of cognitive decline in the Open Access Series of Imaging Studies-3 data set. Among 1098 participants, 274 passed the inclusion criteria (i.e. had at least two cognitive assessments and two amyloid scans). Over 90% of participants were healthy at baseline. Over 8–10 years, some participants progressed to very mild cognitive impairment (*n* = 48), while others stayed healthy (*n* = 226). Participants with cognitive decline show faster amyloid accumulation in the lateral temporal, motor and parts of the lateral prefrontal cortex. These changes in amyloid levels were linked to longitudinal increases in the functional connectivity of select networks, including default mode, frontoparietal and motor components. Our findings advance the understanding of amyloid staging and the corresponding changes in functional organization of large-scale brain networks during the progression of early preclinical Alzheimer’s disease.

## Teaser

Moffat *et al*. found that mild cognitive decline was associated with faster amyloid accumulation in lateral temporal, motor and lateral prefrontal regions. Amyloid changes were associated with increased functional connectivity of default mode, frontoparietal and motor networks, clarifying amyloid staging and its functional correlates in early preclinical Alzheimer’s disease.

## Graphical Abstract

**Graphical abstract:**
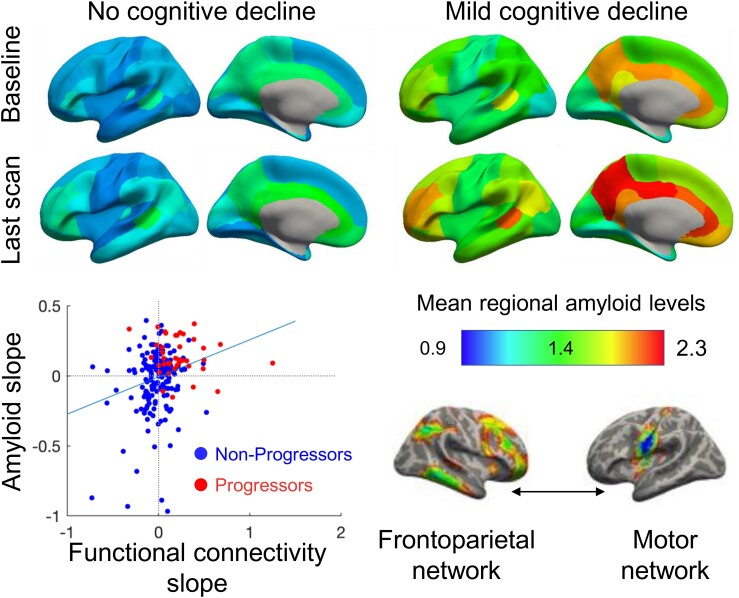

## Introduction

Alzheimer’s disease is a progressive neurodegenerative disease that causes debilitating cognitive impairment. Over 1.5 million deaths globally were attributed to Alzheimer’s disease and other dementias in 2019^1^ with an annual healthcare cost that has surpassed $800 billion.^2^ Attempts to identify new treatments for Alzheimer’s disease have been largely unsuccessful and therefore, there is currently no pharmaceutical intervention that prevents symptoms and further evidence from clinical trials of amyloid-clearing drugs is needed.^3,4^ The inability to treat Alzheimer’s disease-related cognitive symptoms and pathologies has led scientists to turn their focus towards understanding the early accumulation of Alzheimer’s disease pathology.^2^

A defining feature of Alzheimer’s disease is the long ‘preclinical’ phase, during which neuropathology begins to accumulate,^5,6^ but the individual remains cognitively normal. Amyloid beta (Aβ) plaques are thought to appear first^6^ and are detectable via PET scans up to 15 years before the manifestation of cognitive symptoms. Aβ is thought to lead to the emergence or enrichment of other pathologies, such as hyperphosphorylated tau, as the disease progresses.^5,7,8^ Thus, targeting Aβ accumulation in the preclinical stage of Alzheimer’s disease may be an effective strategy for preventing cognitive decline.^5,7,9^

As preclinical Alzheimer’s disease pathology spreads, it affects the functional organization of major brain networks in resting-state functional magnetic resonance (fMRI).^5,10,11^ Accumulation of Aβ and tau affects the highly interconnected neural hubs in particular.^12,13^ Functional changes between brain networks have been observed in the prodromal stages of Alzheimer’s disease, i.e. ‘mild cognitive impairment’ (MCI).^5,10^ Functional disruptions are most frequently reported in the default mode network (DMN) in both MCI and Alzheimer’s disease.^12,14–16^ Extensive evidence shows lower DMN connectivity in Alzheimer’s disease and other dementias,^11,14,17^ although paradoxically, increases have been reported in the preclinical stages.^18^ Thus, the direction of functional connectivity (FC) changes may fluctuate throughout the course of disease progression.^19^ A more comprehensive understanding of functional changes and the timeline at which they occur is needed. Additionally, changes in other brain areas related to executive function and higher cognitive abilities have also been implicated in Alzheimer’s disease, as well as normal aging, including the frontoparietal network (FPN) and cingulo-opercular network.^14,17,20^ Findings from fMRI may direct research into cognitive tests that are sensitive to fMRI changes related to amyloid pathology, in search of multimodal biomarkers.^21^ Further, fMRI network function could help identify whether participants maintain healthy network function and could be targeted by cognitive remediation training or pharmacological interventions.

In this study, we focused on the earliest preclinical stages of Alzheimer’s disease. We aimed to characterize the longitudinal changes of Aβ accumulation and brain network function in older adults who show cognitive decline^9^ within 8–10 years, compared with those who remain cognitively normal over the same time frame. Based on previous attempts to map patterns of Aβ deposition,^22,23^ we hypothesized that mean amyloid levels and rate of amyloid accumulation in the DMN regions would be higher in those who developed cognitive decline compared to those who stayed cognitively normal.^12,15,16^ We also hypothesized significant changes in connectivity in the progressor group compared with the non-progressors in the DMN and FPN.^10,23,24^ Finally, we explore the relationship between longitudinal changes in amyloid and FC across all participants.

## Materials and methods

### Data

Data from the third release of the Open Access Series of Imaging Studies (OASIS-3) were used for analysis.^25^ OASIS-3 is a longitudinal dataset available publicly that includes clinical, cognitive and neuroimaging data from the Washington University Knight Alzheimer Disease Research Centre. The present study utilized resting-state functional MRI, PET imaging for amyloid detection and clinical/demographic data from the OASIS-3 database (https://www.oasis-brains.org/accessed: 2 June 2021). Multiple measures of each data type were taken at different time points over the course of the study, with time frames for a single subject lasting up to 30 years.

### Participants

**Figure 1:**
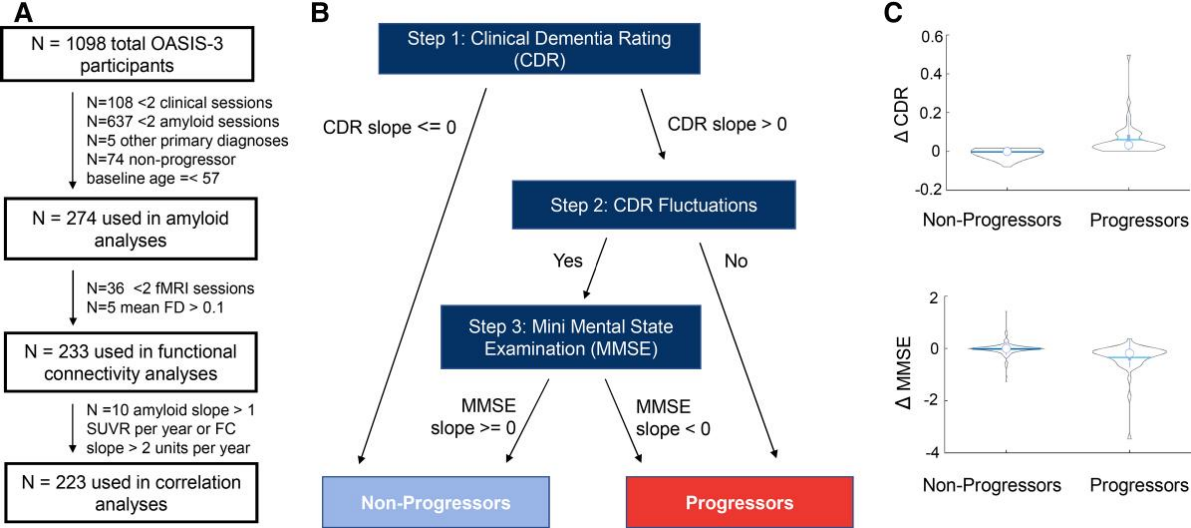
**Classification of participants by progression group.** (**A**) Participants had at least two repeated measures of CDR and MMSE and two PET scans. Participants with primary diagnoses such as Parkinson’s disease were also excluded since we aimed to include participants with very mild cognitive impairment of the Alzheimer’s type. Non-progressors who were younger than 57 years of age at baseline were excluded in order to ensure the progressor and non-progressor groups were age-matched. For the correlation analyses of FC and amyloid levels, we excluded 10 outliers (>5 SD from the mean). The extreme slopes of these outliers are attributed to these participants being scanned at two timepoints very close together (less than a month apart) with different amyloid PET tracers. (**B**) Progressors versus non-progressors were first classified according to the slopes of their CDR scores. For those with increasing CDR slopes, we examined whether the CDR scores fluctuated (please see Supplementary Information for more details). If a participant's score fluctuated more than once, downward MMSE slopes (<0) were used to confirm cognitive decline. Participants with non-increasing CDR scores or unstable fluctuations of CDR scores were classified as non-progressors. (**C**) One-way ANOVA tests showed that CDR and MMSE slopes were significantly different between progressors (*n* = 48) and non-progressors (*n* = 226) (F = 123.27, *P* = 8.45E-20 and F = 40.78, *P* = 7.51E-10 for CDR and MMSE slopes, respectively).

Participants were selected from the OASIS-3 cohort of 1098 subjects. Exclusion criteria were guided by our objective to compare amyloid and FC changes in participants progressing to very mild cognitive impairment versus those who remained healthy. Exclusion criteria included an active psychiatric or neurological disorder, in particular cerebrovascular disease, parkinsonism dementias or frontotemporal dementia. Diagnoses were provided by either a single clinician or based on a consensus diagnosis. Subjects were also excluded if they had <2 amyloid PET scans or clinical sessions (Fig. 1A). The remaining 348 participants were classified as cognitive progressors if they exhibited decline (by a score of 0.5 or more) primarily based on the Clinical Dementia Rating (CDR).^26^ We used linear regressions (*regress.m* function with the number of visits as a predictor variable and the CDR score at each visit as the outcome variable) to compute CDR slopes. We checked whether participants who showed increasing CDR slopes showed any fluctuations in their CDR scores to ensure that progressors show a consistent decline in CDR scores. To resolve fluctuations in CDR scores (i.e. switching between 0 and 0.5 more than once), Mini-Mental State Examination (MMSE)^27^ scores were also examined over time. If CDR scores fluctuated, the slope of MMSE scores had to be <0 to provide confirmation of cognitive decline. Individuals with a baseline score of 0.5 were required to progress to a CDR of 1. Following classification, non-progressors who were younger than 57 years of age at baseline (N = 74) were also excluded to ensure the groups were age-matched, leaving 226 non-progressors and 48 progressors. Fig. 1 shows an overview of the classification process and further details are provided in the Supplementary Materials.

### Amyloid PET acquisition

Amyloid values were acquired via PET scanning with the use of one of two tracers: N-methyl-[11C]2-(4’-methylaminophenyl)-6-hydroxybenzothiazole (PIB) or [^18^F] AV-45 florbetapir. Tracer was controlled for in all analyses using the acquired PET data to account for variance in the detection compound. Values are reported as standardized uptake value ratios (SUVRs) and partial volume correction was completed via the regional spread function in PET Unified Pipeline (PUP) in the Desikan–Killiany atlas^28^ (https://github.com/ysu001/PUP). Amyloid positivity was determined using OASIS3-provided cut-offs (1.19 for AV-45 and 1.42 for PIB with the regional spread function).

### fMRI acquisition

Siemens 3 T scanners were used to obtain blood oxygenation level-dependent signalling data. Resting-state function MRI was used as a measure of FC. During a 6 minute resting-state blood oxygenation level-dependent scan, participants were asked to lay quietly with their eyes open. Most resting-state functional MRI data were collected on Siemens 3T Trio Tim scanner (slices = 36 with a thickness of 4 mm, repetition time TR = 2500 ms, echo time TE = 27 ms, matrix = 64 × 64, overall resolution = 4 mm^3^). On some Siemens 3T Trip Tim scanners, the TR was reduced to 2200 ms. Structural data were pre-processed using the Freesurfer image analysis suite as described in LaMontagne *et al.* 2019 and downloaded from XNAT Central.

### fMRI processing

We kept the processing pipeline as close as possible to the FMRIB’s processing pipeline.^29^ More specifically, we downloaded fMRI data in BIDS format from OASIS and applied the FMRIB statistical library FEAT (v 3.15) for fMRI pre-processing (removing the first three volumes, linear detrending of 100 s, and smoothing with a kernel of 5 mm). We further used MELODIC (Beckmann and Smith, FMRIB Technical Report TR02CB1) to estimate subject-level independent components and applied in-house developed software, Alternative Labelling Tool ^30^ to label these components as signal and noise. Alternative Labelling Tool performance was close to hand classification and reduced mean Framewise Displacement (FD). We regressed out the noise components (with *fsl_regfilt*), thus obtaining de-noised data. All of the above pre-processing occurred in subject space. We further downloaded Freesurfer outputs from OASIS, aiming to leverage the high-quality, nonlinear registration to MNI-152 template available in Freesurfer (*reg-feat2anat*). We mapped each participant’s de-noised data to the MNI-152 space and assessed the registration quality using *slicesidir*. Participants with a mean FD of > 0.1 (after motion correction) were excluded from the analyses to ensure high data quality.

To assess network connectivity, we used a data-driven parcellation derived from 4000 UK Biobank participants that comprised 21 independent components (ICs), resulting in 210 unique connectivity pairs between these ICs. FMRIB statistical library FSLNets was used to generate and z-transform Pearson’s correlations between time series extracted from each IC. We used boundary-based registrations of participants’ de-noised fMRI images to their T1 images and Freesurfer (v 6.1.0) for nonlinear registration between T1 and the MNI-152 space. We used previously published mapping between the IC maps and seven Yeo networks for added interpretation value.^31,32^

### Genotyping

Information on the *APOE* allele was available for all participants in the OASIS-3 data set due to the known effects variations in the gene have on Alzheimer’s disease development.^33^ Genotyping was completed by PCR amplification on the participant’s blood samples. Qiagen, Inc. QIAmp DNA mini blood kits were used in this process. For more information on genotyping, see LaMontagne *et al.*^25^

### Statistical analysis

We aimed to characterize the longitudinal changes of (i) Aβ accumulation and (ii) brain network function across the whole brain in older adults who progress to very mild cognitive impairment (quantified as an increase in CDR scores, Fig. 1) within 8–10 years, compared with those who remain cognitively normal. First, linear mixed effect models assessed the effect of the progression group on amyloid deposition over 8–10-year follow-up period, with a random intercept and a random slope fitted for each subject. Time since baseline (referred to as **time**) was also treated as a random effect (see Supplementary Fig. 1 for plots of residuals versus fitted values). Fixed effects included the baseline CDR score, baseline age, sex, tracer (coded as ‘PIB’ and ‘AV-45’) and time between fMRI and amyloid scans. MATLAB’s *mafdr* function was used to correct for the false discovery rate due to multiple comparisons.

$$amyloid\;SUVR∼1+time^{*}progression\;group+baseline\;CDR+baseline\;age+sex+tracer+time+(1+time\;|Subject)$$

Linear mixed effect models were also used to investigate the effect of progression groups on longitudinal FC. The model used is presented below. These analyses included the mean FD and proportion of signal components as fixed effects in addition to the baseline CDR, baseline age, sex and time between scans. In these analyses, permutation testing using R’s *permlmer* function was used to correct for multiple comparisons (https://rdrr.io/cran/predictmeans/man/permlmer.html).^34^

$$functional\;connectivity\;∼1+time^{*}progression\;group+baseline\;CDR+baseline\;age+sex+prop\;signal+mean\;fd+time+(1+time\;|Subject)$$

Slopes of amyloid accumulation and change in FC were calculated as annual rates of change. Using the linear mixed effect model defined above, we calculated the difference between each participant’s model-fitted baseline and the last amyloid SUVR measurement. We then divided this number by the number of years between the baseline and last PET scan to compute the slope of deposition. For change in connectivity between functional brain networks, we similarly used the model-fitted functional connectivities between ICs and calculated the difference between the values obtained from the first and last fMRI scan, divided by the time between scans.

$$amyloid\;slopes=\frac{last\;amyloid\;SUVR-baseline\;amyloid\;SUVR\;}{years\;between\;baseline\;and\;last\;amyloid\;PET\;scans\;}$$

$$functional\;connectivity\;(FC)\;slopes=\frac{last\;FC\;-\;baseline\;FC\;}{years\;between\;baseline\;and\;last\;rs-fMRI\;scans\;}$$

We then identified the amyloid slopes from 18 brain regions that demonstrated faster rates of amyloid accumulation in progressors than in non-progressors (FDR-corrected *P* < 0.05; Supplementary Table 3). Significant interactions between time and progressor group indicate a different pattern of amyloid beta accumulation in progressors versus non-progressors. These slopes were analysed using principal component analysis (PCA). The PCA1 scores were correlated with the slopes of 23 functional connectivities that also showed a significant time × progression group interaction to explore the association between longitudinal amyloid accumulation and longitudinal changes in FC. We corrected for multiple comparisons of the amyloid-FC slope correlations using the Benjamini and Hochberg method using MATLAB’s *mafdr* function.^35^

### Data/code availability

Data used in this article were obtained from the OASIS-3 database (https://www.oasis-brains.org/) and are freely available after registration. The code used for analysis is available at https://github.com/gemmamoffat/OASIS3_LMEanalyses.

## Results

### Study participants

Demographic information from the 274 participants remaining following exclusions is presented in Table 1. Progression groups did not differ by age, gender, education, baseline CDR, *APOE* status, race or ethnicity. As expected, there were significant group differences in the CDR slope and MMSE slope, confirming cognitive decline in the progressor group. Most progressors (*n* = 43) started at CDR-0 and progressed to CDR-0.5, thus developing very mild cognitive impairment.^36,37^ A total of 27 participants crossed the threshold for amyloid positivity between baseline and final follow-up, with the proportion of amyloid positive progressors remaining significantly greater than amyloid positive non-progressors (Table 1). Given that we excluded participants with conditions other than Alzheimer’s disease, we argue that our sample is likely assessing the very early stages of the Alzheimer’s disease type of dementia. There was also a significant difference in the average length of time spent in the study, with the progressors being observed on average for ∼2 years longer than the non-progressors.

**Table 1:** Participant demographics. Mean values (SDs) are shown

	Non-Progressors	Progressors	Statistical Significance (*P*-value)
**Number of subjects**	226	48	
**Age at baseline**	67.42 (SD 6.46)	69.33 (SD 8.20)	0.0788
**Gender (male/female)**	90/136	18/30	0.7649
**Education**	15.85 (SD 2.50)	15.13 (SD 2.48)	0.0703
**CDR slope**	0	0.0607(SD 0.082)	8.45*10^−24^
**% Baseline CDR = 0**	94%	90%	0.3388
**MMSE Slope**	0	−0.345 (SD 0.580)	7.5*10^−10^
**Length of time in study**	8.16 (SD 3.76)	10.42 (SD 5.35)	5.7*10^−4^
*APOE* status (e2*/e3e3/e3e4 or e4e4)	39/126/61	8/22/18	0.4819
**Race (Caucasian/African American/Asian)**	202/23/1	45/3/0	0.6251
**Ethnicity (Non-Hispanic/Hispanic)**	225/1	48/0	0.6443
**Amyloid-β+ baseline**	46 (20%)	25 (53%)	1.0*10^−5^
**Amyloid-β+ last follow-up**	73 (32%)	27 (56%)	0.002
**Number of amyloid follow-up visits**	2.65 (SD 0.98)	2.77 (SD 0.99)	0.4094
**Number of MRI follow-up visits**	3.15 (SD 1.01)	3.14 (SD 1.05)	0.9242

CDR, clinical dementia rating; MMSE, mini-mental state examination. Average age, education, CDR and MMSE slopes (per year) and length of time in study are shown along with SD. For categorical comparisons, we used χ^2^ test, while for continuous variables, we used a one-way analysis of variance.

### Higher levels and faster accumulation of amyloid in progressors than non-progressors

**Figure 2:**
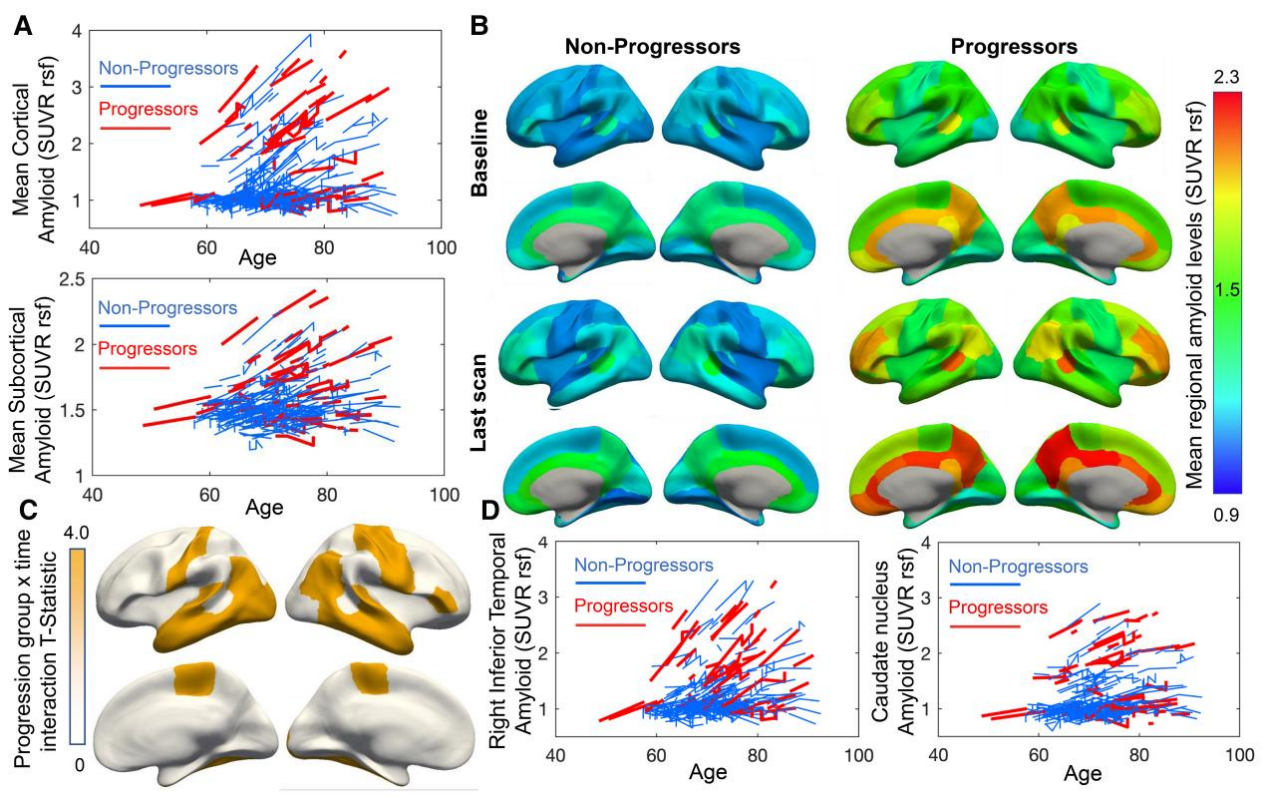
**Higher amyloid levels and faster amyloid deposition in progressors versus non-progressors.** (**A**) Spaghetti plots of the linear mixed effect model-fitted amyloid SUVR levels show higher mean cortical and subcortical amyloid SUVR levels in progressors compared with non-progressors. Accumulation rates for mean cortical and subcortical amyloid levels did not differ significantly between groups. (**B**) Mean model-fitted amyloid values are shown in each of the 68 cortical regions from the Desikan–Killiany atlas. (**C**) Visualized statistically significant (false discovery rate-corrected P < 0.05) regions for a progression group × time interaction. (**D**) Compared with non-progressors, progressors show steeper slopes of amyloid accumulation in the right inferior temporal gyrus but not in the caudate nucleus as example regions.

**Figure 3:**
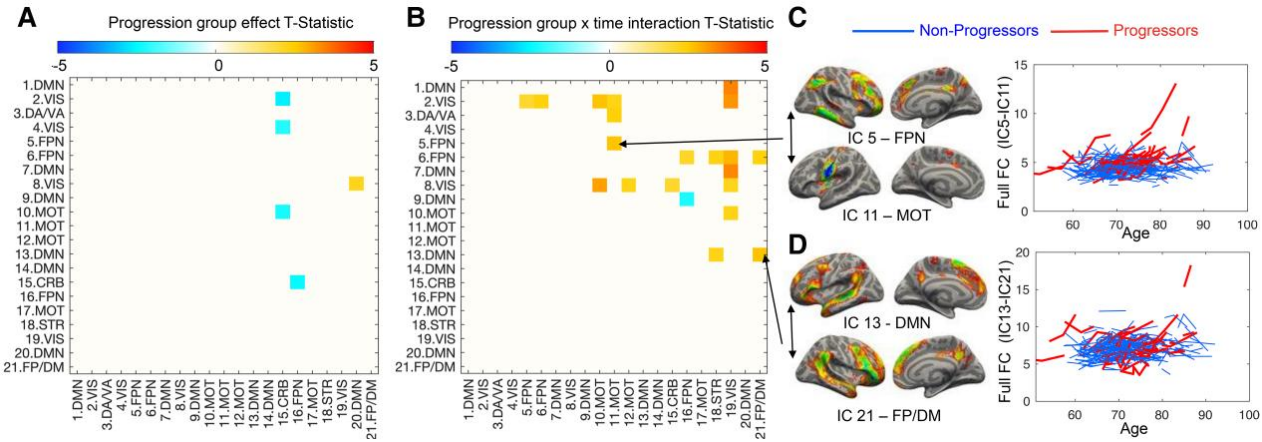
**FC is lower and increases faster over time in progressors versus non-progressors.** (**A**) Progressors showed reduced connectivity in 5 pairs of ICs, including primarily visual and cerebellar regions. (**B**) Progressors also showed faster rates of increase in FC among FPN, default mode (DMN) and motor regions. For example, progressors show steeper individual trajectories of IC-5 with IC-11 **(C)** and IC-13 with IC-21 **(D)** than non-progressors.

Using mixed effect linear models, we found a statistically significant difference in mean cortical (beta = 0.43, SE = 0.087, T = 4.96, *P* = 8.66*10^−7^) and subcortical (beta = 0.13, SE = 0.036, T = 3.66, *P* = 0.0003) amyloid levels between progressors and non-progressors (Fig. 2B, Supplementary Table 1A, B and Figs 2, 3). By-region analyses showed that these main effects were significant after FDR correction (*P*_FDR_<0.05) for all 34 Freesurfer cortical regions in both hemispheres and all nine subcortical regions studied (Fig. 2A and B, Supplementary Table 2). Moreover, progressors showed higher rates of amyloid positivity compared to non-progressors (Table 1, baseline χ^2^ = 20.76, *P* < 0.001, last follow-up χ^2^ = 9.80, *P* = 0.002). This finding suggests that over 50% of our progressor group may be at risk for Alzheimer’s disease due to elevated amyloid followed by the development of subtle cognitive symptoms.^38^

Further, we found a statistically significant interaction between the progression group and time from baseline assessment, indicating a greater rate of amyloid increase over time in progressors compared to non-progressors in lateral and inferior temporal, motor (postcentral gyrus) and inferior frontal regions (Fig. 2C, Supplementary Table 2) after FDR correction. However, no significant interaction was present in mean cortical (beta = 0.014, SE = 0.008, T = 1.65, *P* = 0.099) or subcortical amyloid (beta = −0.004, SE = 0.005, T = −0.82, *P* = 0.41) (Supplementary Table 1A and B). Progressors showed faster accumulation of amyloid in lateral temporal and motor regions. No significant interactions were found in subcortical regions (*P*_FDR_ > 0.1). We showcase the longitudinal amyloid trajectories for the right inferior temporal lobe, the region with the strongest interactive effect, in Fig. 2D. For comparison, we also show amyloid trajectories for the caudate nucleus (Fig. 2D), one of the subcortical regions that do not show a difference in the rate of deposition based on the group.

### Functional connectivity is lower and increases faster over time in progressors but not in non-progressors

**Figure 4:**
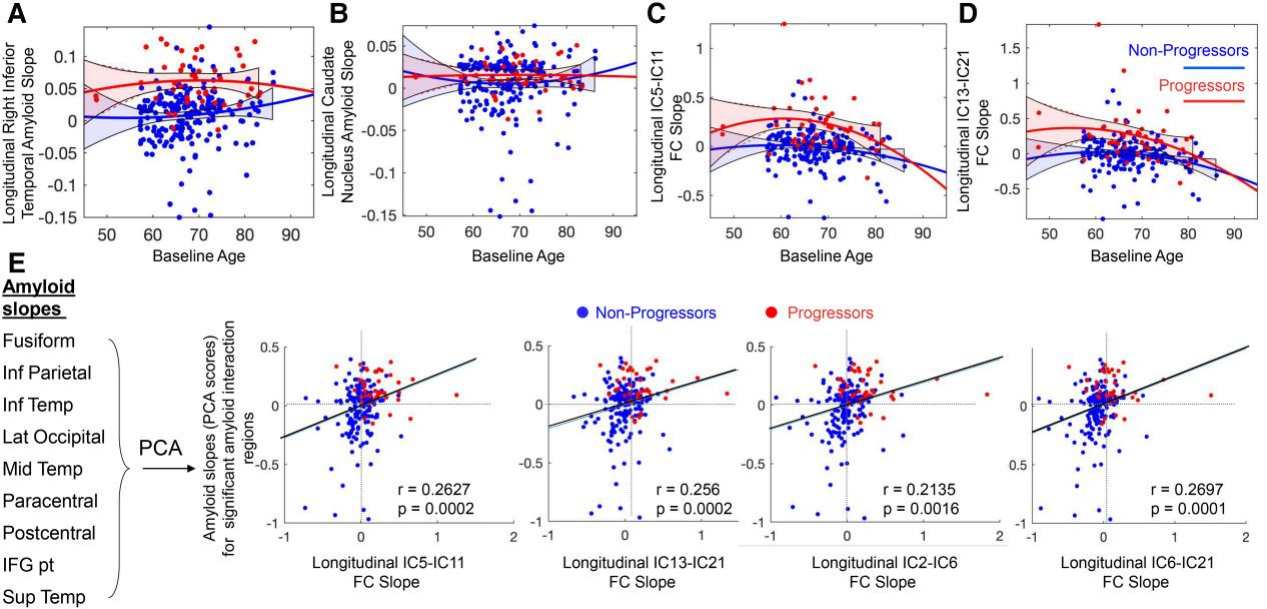
**Correlation between longitudinal changes in amyloid accumulation and FC.** Quadratic trajectories and 90% confidence intervals (calculated using MATLAB’s *polyconf* function) are shown in **A-D**. We excluded 15 amyloid slopes that were derived from participants with very short follow-up duration and deviated >4 standard deviations from the mean of all amyloid slopes. Amyloid deposition slopes (derived from model-fitted values as described in the Materials and methods) were higher in progressors than non-progressors in the right inferior temporal lobe (**A**) but not in the caudate nucleus (**B**). FC slopes (derived from model-fitted values) are plotted against baseline age for two example connectivities between IC-5 and IC-11 (FPN-motor network, **C**) and IC-13 and IC-21 (DMN-FP/DMN, **D**), respectively. (**E**) Visualized correlations between FC slopes and amyloid PCA scores for regions with statistically significant progression group x time interactions.

We found significant reductions in FC in progressors compared with non-progressors localized to visual, cerebellar and FPN ICs (Fig. 3A, Supplementary Table 3). We also found a significant interaction between progression group and time, suggesting that the rate of increase in FC of visual, motor, DMN and FPN ICs was faster in progressors than non-progressors (Fig. 3B–D and Fig. 4C and D, Supplementary Table 4). For comparison, we show the longitudinal amyloid trajectories in Fig. 4A and B.

### Amyloid accumulation was associated with increased functional connectivity driven by higher slopes in the progressor group

We found that amyloid accumulation slopes in all regions with a significant interaction were strongly correlated and summarized by the first principal component of the PCA (PCA1), which explained 99% of the variance in amyloid slopes. Further, we found moderate but significant positive correlations between PCA1 scores and the slopes of functional connectivities that showed a significant group × time interaction as well. (Fig. 4). Participants with faster amyloid deposition (primarily progressors, shown in red) also showed increases in FC between FPN (IC-5) and motor network components (IC-11) and between IC-13 and IC-21 (r = 0.26, *P*_FDR_ = 0.0002 and r = 0.26, *P*_FDR_ = 0.0002, respectively, Supplementary Table 5), both components encompassing lateral temporal and superior frontal gyri. Components with a significant group × time interaction encompassed some of the same brain areas that showed a significant interaction in the amyloid linear mixed effect models (i.e. inferior temporal, pre- and postcentral and inferior frontal gyri). As a result, the correlation between amyloid and FC slopes appears to be driven by higher slopes of both variables in the progressor group. Significant correlations were also found for visual regions (*P* < 0.001) and are summarized in Supplementary Table 5. We present a mapping between the Desikan–Killiany parcellation that was used in the amyloid analyses and the ICs in Supplementary Fig. 4. Briefly, components IC-11, IC-2, IC-3, IC-5 and IC-13 showed the most overlap with the regions that showed a significant group × time interaction on amyloid levels.

### Sensitivity analyses

We have conducted several sensitivity analyses. First, we excluded participants with a CDR-0.5 at baseline (*n* = 5, Supplementary Table 2); second, we excluded participants who had a CDR-0 at final follow-up (*n* = 14, Supplementary Table 2); third, we added *APOE* as a covariate to amyloid models (Supplementary Table 2); fourth, we added demographics × time interactions (Supplementary Table 2) to amyloid models; fifth, we added education and amyloid to FC models (Supplementary Tables 3 and 4). These sensitivity analyses showed that our findings were robust, with more details presented in the Supplementary Materials.

## Discussion

In this study, we aimed to characterize patterns of regional amyloid accumulation and FC changes between brain networks in participants with very mild cognitive decline. As expected, the progressor group experienced a decline in cognitive status over a period of 8–10 years, which was determined by changes in CDR and MMSE scores. Using longitudinal *in-vivo* amyloid data, we show that the progressors who develop very mild cognitive impairment (CDR-0.5) show significantly faster amyloid accumulation in lateral and inferior temporal, motor (postcentral gyrus) and inferior frontal regions. Further, progressors also showed greater increases in FC among many of the same temporal, motor and inferior frontal cortex regions, especially in ICs IC-11, IC-2, IC-3, IC-5 and IC-13. Previous research suggests that Aβ plaque accumulation in preclinical Alzheimer’s disease may have functional correlates.^19,39–41^ When focusing on amyloid and functional connectivities in regions with faster rates of change in progressors than non-progressors, we also found that rates of amyloid accumulation were correlated with rates of change in FC. Our results contribute to the understanding of amyloid staging in the earliest phases of preclinical Alzheimer’s disease and show that changes across a number of functional brain networks are linked to amyloid accumulation.

Since the presence of Aβ plaques was initially associated with Alzheimer’s disease pathology,^42^ there have been many attempts to map amyloid accumulation and establish stages of amyloid spread in the brain. Our results are consistent with several amyloid staging models^8,43,44^ which pinpoint areas across the neocortex as the first locations to develop sparse Aβ plaque accumulation. According to these models, subcortical regions along with the posterior cingulate develop Aβ deposits later in the preclinical phase, closer to when cognitive symptoms begin to appear. Our preclinical progressors did not show significantly faster accumulation in the posterior cingulate or subcortical regions, supporting the above amyloid staging model.^8^ These findings are in contrast with other proposed amyloid staging models^45^ however, which suggest that the precuneus, posterior cingulate and medial temporal regions are the initial locations of increased Aβ. We argue that the discrepancy between findings may be due to differences in disease staging (i.e. the extent of progression of Alzheimer’s disease pathology). A major strength of this study is that even the participants in our cognitive progression group most often only exhibited a slight decline in cognitive performance (CDR-0.5), which occurs prior to the onset of MCI^36,37^ suggesting that our findings are ideal for clarifying initial preclinical amyloid staging. Over 50% of participants in our progressor group were amyloids positive, consistent with previous studies of preclinical Alzheimer’s disease ^46^ and MCI,^47^ which included both amyloid positive and amyloid-negative individuals. While typical preclinical Alzheimer’s disease is characterized by asymptomatic cerebral amyloidosis,^5^ including amyloid-negative participants with longitudinal cognitive decline is valuable as it allows us to assess amyloid accumulation in individuals with varying levels of amyloid burden. Further, some studies suggest that the earliest accumulation of amyloid-β occurs in the DMN and concurrently affects brain connectivity.^48^ However, this finding was in amyloid-β accumulators and was not based on cognitive staging, unlike in the present study. Cognitive staging is critical in assessing Alzheimer’s disease^38^ and can lead to different results, as evidenced by our findings. A replication of our analyses in a sample population with a larger proportion of progressors would be beneficial to support our results, as a potential limitation of the present study is the imbalance between our group sample sizes.

Neuronal toxicity due to Aβ plaques has been hypothesized to cause the decreases in brain network FC that are repeatedly observed in the DMN of Alzheimer’s disease patients^14–16^ and cognitively normal older adults.^11,14,23,49,50^ The DMN has also been implicated in having significant functional disturbance due to age, even in the healthy elderly,^51^ and these effects are thought to be enhanced by the deposition of Aβ proteins resulting in reorganization of neural pathways.^41,50^ This evidence is consistent with our cross-sectional findings of strongly elevated amyloid levels and overall reduced FC in temporal, motor and visual regions in progressors. As expected, we found increases in FC that occur over a 10-year follow-up as participants move from normal ageing to very mild cognitive decline. These increases in FC in visual, motor, default mode and FPN may be compensatory.^14,24,52,53^ The Aβ plaques and other Alzheimer’s disease pathology, such as tau neurofibrillary tangles, may disrupt connections between brain regions and functional reorganization may help make up for these losses. Indeed, among patients with MCI but not in healthy controls, increased connectivity of regions such as the precentral and middle frontal gyri is associated with better cognitive performance.^52^ However, the reorganization of an Aβ-disrupted brain may be maladaptive.^54^ As the brain makes momentarily stronger connections to replace those that are pathologically altered, aberrant hyperconnectivity could be contributing to the memory deficits and cognitive symptoms of Alzheimer’s disease. Moreover, hyperconnectivity between functional networks would help facilitate Aβ spread as FC changes precede Aβ accumulation^11,49^ and functional networks have been shown to facilitate tau spread.^19,55,56^

A recent study of the OASIS-3 dataset^57^ found that fMRI data did not aid in the prediction of future cognitive decline. This study used baseline structural/functional/non-brain data to form a predictive model that estimates future cognitive status. We propose that the identified lack of utility of FC data in this case may be due to the dynamic changes that appear to occur within and between brain network as Alzheimer’s disease progresses and molecules like Aβ and tau accumulate.^19,58,59^ There is great variability in findings on FC changes in preclinical Alzheimer’s disease,^11,14,20^ which has been hypothesized to be due to inconsistent compensation mechanisms based on the state of structural brain disruption.^20^ Using longitudinal data to look more thoroughly at fluctuations in brain networks changes, as we did in this study, is important to track the timeline of modifications that occur and establish where exactly along the disease continuum that these changes originate.^60–62^ The socio-demographic diversity of study populations may also lead to inconsistencies in the fMRI literature to date. The risk and burden of Alzheimer’s disease are greater among women compared to men. Moreover, African American/Black and Hispanic/Latino individuals are more vulnerable compared with non-Hispanic white individuals. Disparities in FC and amyloid spread as a function of sex and race are an unexplored potential mechanism behind risk and burden disparities in study populations.^63,64^ In the future, including tau trajectories as well as other structural and pathological changes such as neuroinflammation that appears to occur early in Alzheimer’s disease will help assess the overall aetiology of preclinical disease and conclusively determine driving factors for the dynamic changes in FC.

Some of the regions we identified as significant in preclinical amyloid accumulation have also been shown to accumulate tau in Alzheimer’s disease.^65,66^ While Amyloid-β and tau follow different trajectories, Amyloid-β triggers the conversion of tau from a normal to a toxic state^67^ and facilitates tau spread from the locus coeruleus and entorhinal cortex to the cortex. Tau may also enhance Amyloid-β toxicity via the feedback loop.^68^ Tau trajectories are predicted to disrupt the lateral and inferior temporal (as well as medial temporal) regions in stages I/II and III/IV before spreading more globally across the cortex from stage V onwards.^65,66^ It has also been found that increased amyloid burden may serve as a predictor for increased tau accumulation, suggesting that as Alzheimer’s disease progresses, Aβ promotes and triggers tau spread and hyperphosphorylation.^8^ Our localization of significant amyloid deposition to the temporal lobe, specifically the lateral and inferior temporal cortices, aligns with this hypothesis by providing evidence that early Aβ accumulation occurs in the same areas where tau is predicted to begin its cortical spread.

The validity of amyloid beta plaques as a target for Alzheimer’s disease treatment has been widely debated. Nevertheless, multiple amyloid targeting treatment trials are underway. One treatment, aducanumab, has Food and Drug Administration approval. The treatment is administered as an anti-amyloid antibody and is recommended for early stage Alzheimer’s disease.^9,69,70^ Due to the frequency of Aβ positivity in normal aging, it has previously been suggested that accelerated rates of Aβ accumulation may help in predicting preclinical Alzheimer’s disease rather than mean amyloid values themselves.^22^ Importantly, differences in the regional distribution of amyloid may also help to distinguish preclinical from benign Aβ deposition.^71^ Our study shows that faster Aβ deposition in lateral temporal and motor regions is evident in preclinical progressors.

To summarize, we report higher cortical amyloid levels and faster accumulation rates specific to the lateral and inferior temporal, inferior parietal cortices and pre- and postcentral gyri of those who develop cognitive decline. We also report increases in FC in default mode and FPN in the visual and motor regions in those who develop cognitive decline. This characterization of brain changes in the earliest stages of Alzheimer’s disease contributes to our understanding of amyloid staging, which is critical to diagnosing and treating patients before the pathological processes become irreversible.

## Supplementary Material

fcac282_Supplementary_Data

## Abbreviations

*APOE* =: apolipoprotein E gene
CDR =: clinical dementia rating
DMN =: default mode network
FC =: functional connectivity
FD =: framewise displacement
FDR =: false discovery rate
fMRI =: functional magnetic resonance imaging
FPN =: frontoparietal network
IC =: independent component
MCI =: mild cognitive impairment
MELODIC =: multivariate exploratory linear optimized decomposition into independent components
MMSE =: mini-mental state examination
MNI152 =: montreal neurological institute standard space template
OASIS =: open access series of imaging studies
PCA =: principal component analysis
PET =: positron emission tomography
PIB =: pittsburgh compound B
PUP =: PET unified processing
SD =: standard deviation
SE =: standard error
SUVR =: standardized uptake value ratio
