# Supplementary material for: Unravelling the relationship between amyloid accumulation and brain network function in normal aging and very mild cognitive decline: a longitudinal analysis
Source: Brain Commun. 2022 Nov 2;4(6):fcac282. doi: 10.1093/braincomms/fcac282 (PMC9678202; doi:10.1093/braincomms/fcac282)
Supplement: fcac282_Supplementary_Data [file fcac282_supplementary_data.docx]

**Supplementary Materials**

**Sample characteristics**

At baseline, 263 (96%) participants were scanned with the PIB tracer, while 11 (4%) participants were scanned with AV-45. At the last follow-up, 137 (50%) participants were scanned with PIB while 137 (50%) participants were scanned with AV-45.

On average, progressors had a mean of 9.75 follow-up visits for CDR (range 3 - 29) while non-progressors had a mean of 7.55 follow-up visits for CDR (range 2 - 21).

There were 19 participants who achieved a CDR score >0.5 at baseline, 5 of whom were progressors as they later showed a CDR score of 1, and 14 participants who were categorized as non-progressors.

**Progressor vs Non-progressor Classification Examples**

For example, a participant who scored 0, 0, 0, 0.5, 0, 0.5 on their six visits would be classified as a progressor because they showed an increase in CDR scores and only showed one fluctuation.

Participants who scored e.g. 0, 0.5, 0, 0.5, 0, 0.5 on their six visits would be only classified as a progressor if they also showed a decrease in their MMSE scores since they showed more than one fluctuation in their CDR score. There were 11 progressors who showed at least two fluctuations as described above and decreases in their MMSE scores were used to confirm cognitive decline.

Finally, some participants (n= 14) reverted to 0 on their last assessment (e.g. 0, 0, 0, 0.5, 0.5, 0). They were classified as progressors since they showed a consistent increase in CDR scores. We re-analysed the data while excluding the abovementioned participants.

**Sensitivity analyses**

We have reanalysed the data five times in order to assess the robustness of our results.

1. Excluding participants with a starting score of CDR-0.5

First, we have excluded CDR-0.5 participants (n=5). This reanalysis did not affect our main findings of longitudinal amyloid trajectories as shown in the Supplementary Table 2.

2. Excluding participants with a final score of CDR-0

Second, we have re-analysed the data while excluding progressors who had a CDR of 0 at their final follow-up. The results of the reanalysis are shown in Supplementary Table 2. Excluding these 14 participants reduced power and rendered some of our effects non-significant, although the strongest effects (in superior temporal, inferior temporal, inferior parietal, middle temporal, fusiform gyri) remain significant (P_FDR_<0.05) despite the decreased sample size.

3. Adding APOE status as a covariate

Third, we have added APOE as a covariate to amyloid models:

$$amyloid SUVR\sim1+\boldsymbol{time*progression group}+baseline CDR+APOE+baseline age+sex+tracer+time+\left( 1+time \right|Subject)$$

This reanalysis strengthened the effects as shown in Supplementary Table 2.

4. Covarying for time x demographics interactions

Fourth, we covaried for time x baseline age and time x sex interactions. Including these interactive terms did not substantially alter the effects reported in the main results section. This reanalysis slightly strengthened the effects as shown in Supplementary Table 2.

5. Covarying for education and amyloid in FC analyses

Finally, we have added education as a covariate to FC models. The results of this reanalysis are shown in Supplementary Tables 3 and 4. Covarying for education did not substantially alter our findings. Similarly, covarying for baseline amyloid in FC models did not substantially alter our findings.

Supplementary Figure 1. **Residual vs. Fitted Values plots.** Visualization of the residual values versus fitted values from the linear mixed models used for **a)** mean cortical and **b)** mean subcortical amyloid accumulation.

Supplementary Table 1a. Linear mixed effect model results for mean cortical amyloid levels (SUVR rsf). Degrees of freedom = 272.

|  |  | **Beta** | **SE** | **T-Statistic** | **P-Value** |
| --- | --- | --- | --- | --- | --- |
|  | Intercept | 0.1364 | 0.32305 | 0.42217 | 0.67302 |
|  | Tracer | -0.232 | 0.014928 | -15.543 | 2.11E-47 |
|  | Time between scans | 0.0458 | 0.003647 | 12.546 | 6.78E-33 |
|  | **Progression group** | **0.4312** | **0.086899** | **4.9618** | **8.66E-07** |
|  | Baseline age | 0.0148 | 0.0048065 | 3.0808 | 0.0021406 |
|  | Baseline CDR | 0.525 | 0.23131 | 2.2699 | 0.023499 |
|  | Sex | 0.0227 | 0.06631 | 0.3423 | 0.73222 |
|  | Progression group*time | 0.0136 | 0.0082668 | 1.6504 | 0.099287 |

Supplementary Table 1b. Linear mixed effect model results for mean subcortical amyloid levels (SUVR rsf). Degrees of freedom = 272.

|  | **Beta** | **SE** | **T-Statistic** | **P-Value** |
| --- | --- | --- | --- | --- |
| Intercept | 1.1374 | 0.1253 | 9.0792 | 9.62E-19 |
| Tracer | -0.0681 | 0.0121 | -5.619 | 2.71E-08 |
| Time between scans | 0.0179 | 0.0023 | 7.6851 | 4.82E-14 |
| **Progression group** | **0.1307** | **0.0357** | **3.6638** | **0.0003** |
| Baseline age | 0.005 | 0.0019 | 2.7106 | 0.0069 |
| Baseline CDR | 0.1551 | 0.0875 | 1.7722 | 0.0768 |
| Sex | 0.0232 | 0.0254 | 0.9152 | 0.3604 |
| Progression group*time | -0.0041 | 0.005 | -0.8176 | 0.4139 |

Supplementary Table 2. Amyloid accumulation by region (Desikan-Killiany atlas)**.** Degrees of freedom = 272. T-statistics (T), uncorrected P-values (P) and FDR-corrected P-values (P_FDR_) are shown for the progression group and the progression group x time interaction. For reanalysis 1 (R1), we excluded participants who had CDR of 0.5 or higher at baseline. For reanalysis 2 (R2), progressors with CDR = 0 at last follow-up were excluded. For reanalysis 3 (R3), we included APOE status (e2 carrier, e3/e3 carrier, e3/e4 carrier, e4/e4 carrier). For reanalysis 4 (R4), we included demographics x time (age x time, sex x time) interactions. Significant interactions (after FDR correction) are highlighted with an outline.

| **Region** | **Group T** | **Group P (uncorr.)** | **Group P_FDR_** | **Group x Time T** | | **Group x Time P** | | **Group x Time P_FDR_** | | **Group x Time P_FDR_**  **R1** | | **Group x Time P_FDR_**  **R2** | | **Group x Time P_FDR_**  **R3** | | **Group x Time P_FDR_ R4** | |  |
| --- | --- | --- | --- | --- | --- | --- | --- | --- | --- | --- | --- | --- | --- | --- | --- | --- | --- | --- |
| **L SSTSBANK** | 4.61 | 4.77E-06 | 7.94E-07 | | 1.74 | | 0.083 | | 0.094 | | 0.205 | | 0.091 | | 0.082 | | 0.084 | |
| **L CAUDANTCNG** | 5.29 | 1.60E-07 | 1.22E-07 | | -0.81 | | 0.419 | | 0.247 | | 0.206 | | 0.459 | | 0.185 | | 0.213 | |
| **L CAUDMIDFRN** | 4.89 | 1.22E-06 | 3.59E-07 | | 1.61 | | 0.108 | | 0.113 | | 0.208 | | 0.156 | | 0.081 | | 0.086 | |
| **L CUNEUS** | 0.72 | 0.4730116 | 0.0244684 | | 0.83 | | 0.405 | | 0.255 | | 0.220 | | 0.574 | | 0.195 | | 0.211 | |
| **L ENTORHINAL** | 1.89 | 0.0587354 | 0.0034066 | | -0.83 | | 0.407 | | 0.251 | | 0.226 | | 0.445 | | 0.188 | | 0.218 | |
| **L FUSIFORM** | 3.32 | 0.0009554 | 7.03E-05 | | 2.78 | | 0.006 | | 0.016 | | 0.035 | | 0.055 | | 0.014 | | 0.011 | |
| **L INFRPRTL** | 4.04 | 5.79E-05 | 5.83E-06 | | 3.03 | | 0.003 | | 0.014 | | 0.036 | | 0.032 | | 0.011 | | 0.011 | |
| **L INFRTMP** | 3.49 | 0.0005113 | 3.99E-05 | | 3.71 | | 0.000 | | 0.002 | | 0.005 | | 0.011 | | 0.002 | | 0.001 | |
| **L ISTHMUSCNG** | 4.31 | 1.84E-05 | 2.27E-06 | | 0.28 | | 0.783 | | 0.322 | | 0.366 | | 0.583 | | 0.261 | | 0.275 | |
| **L LATOCC** | 1.35 | 0.1774166 | 0.009702 | | 2.25 | | 0.025 | | 0.039 | | 0.037 | | 0.055 | | 0.033 | | 0.032 | |
| **L LATORBFRN** | 4.79 | 1.97E-06 | 4.70E-07 | | 0.09 | | 0.925 | | 0.354 | | 0.372 | | 0.580 | | 0.280 | | 0.305 | |
| **L LINGUAL** | 2.83 | 0.0047276 | 0.0003232 | | 0.33 | | 0.742 | | 0.319 | | 0.295 | | 0.595 | | 0.250 | | 0.278 | |
| **L MEDORBFRN** | 4.71 | 3.00E-06 | 5.73E-07 | | -0.47 | | 0.642 | | 0.298 | | 0.253 | | 0.569 | | 0.235 | | 0.258 | |
| **L MIDTMP** | 4.17 | 3.37E-05 | 3.79E-06 | | 2.71 | | 0.007 | | 0.016 | | 0.033 | | 0.035 | | 0.014 | | 0.013 | |
| **L PARAHPCMPL** | 3.54 | 0.0004219 | 3.36E-05 | | -0.19 | | 0.851 | | 0.330 | | 0.335 | | 0.451 | | 0.248 | | 0.283 | |
| **L PARACNTRL** | 3.84 | 0.0001318 | 1.20E-05 | | 2.87 | | 0.004 | | 0.015 | | 0.035 | | 0.055 | | 0.013 | | 0.009 | |
| **L PARSOPRCLRS** | 5.31 | 1.46E-07 | 1.40E-07 | | 0.76 | | 0.445 | | 0.247 | | 0.299 | | 0.448 | | 0.211 | | 0.216 | |
| **L PARSORBLS** | 4.14 | 3.90E-05 | 4.26E-06 | | 0.41 | | 0.679 | | 0.305 | | 0.304 | | 0.608 | | 0.248 | | 0.264 | |
| **L PARSTRNGLRS** | 5.36 | 1.11E-07 | 1.41E-07 | | 1.09 | | 0.278 | | 0.197 | | 0.226 | | 0.390 | | 0.171 | | 0.175 | |
| **L PERICLCRN** | 2.81 | 0.0050143 | 0.0003309 | | 1.34 | | 0.179 | | 0.154 | | 0.200 | | 0.257 | | 0.123 | | 0.138 | |
| **L POSTCNTRL** | 3.95 | 8.70E-05 | 8.12E-06 | | 2.72 | | 0.007 | | 0.017 | | 0.033 | | 0.059 | | 0.013 | | 0.012 | |
| **L POSTCNG** | 5.11 | 4.06E-07 | 1.73E-07 | | 0.53 | | 0.596 | | 0.291 | | 0.335 | | 0.612 | | 0.227 | | 0.236 | |
| **L PRECNTRL** | 4.81 | 1.80E-06 | 4.60E-07 | | 2.09 | | 0.037 | | 0.052 | | 0.098 | | 0.141 | | 0.045 | | 0.037 | |
| **L PRECUNEUS** | 4.91 | 1.13E-06 | 3.59E-07 | | 1.58 | | 0.116 | | 0.117 | | 0.200 | | 0.344 | | 0.089 | | 0.085 | |
| **L ROSANTCNG** | 5.83 | 8.39E-09 | 3.21E-08 | | -0.69 | | 0.491 | | 0.258 | | 0.230 | | 0.554 | | 0.191 | | 0.225 | |
| **L ROSMIDFRN** | 5.23 | 2.21E-07 | 1.21E-07 | | 1.38 | | 0.169 | | 0.150 | | 0.221 | | 0.297 | | 0.119 | | 0.132 | |
| **L SUPERFRN** | 5.51 | 5.00E-08 | 9.56E-08 | | 1.28 | | 0.201 | | 0.168 | | 0.230 | | 0.242 | | 0.129 | | 0.134 | |
| **L SUPERPRTL** | 4.76 | 2.38E-06 | 5.06E-07 | | 1.50 | | 0.133 | | 0.126 | | 0.163 | | 0.321 | | 0.098 | | 0.084 | |
| **L SUPERTMP** | 4.10 | 4.55E-05 | 4.71E-06 | | 2.85 | | 0.005 | | 0.014 | | 0.033 | | 0.051 | | 0.013 | | 0.013 | |
| **L SUPRAMRGNL** | 4.82 | 1.74E-06 | 4.76E-07 | | 1.23 | | 0.218 | | 0.171 | | 0.228 | | 0.292 | | 0.132 | | 0.140 | |
| **L FRNPOLE** | 3.48 | 0.0005274 | 4.04E-05 | | 0.78 | | 0.434 | | 0.246 | | 0.309 | | 0.454 | | 0.203 | | 0.219 | |
| **L TMPPOLE** | 2.74 | 0.0062874 | 0.0004011 | | 0.01 | | 0.994 | | 0.366 | | 0.355 | | 0.510 | | 0.292 | | 0.306 | |
| **L TRANSTMP** | 3.41 | 0.0006941 | 5.21E-05 | | 0.31 | | 0.759 | | 0.321 | | 0.369 | | 0.585 | | 0.250 | | 0.281 | |
| **L INSULA** | 4.20 | 2.98E-05 | 3.46E-06 | | 0.67 | | 0.504 | | 0.260 | | 0.230 | | 0.485 | | 0.224 | | 0.227 | |
| **R SSTSBANK** | 4.29 | 2.04E-05 | 2.44E-06 | | 1.75 | | 0.080 | | 0.099 | | 0.113 | | 0.106 | | 0.084 | | 0.075 | |
| **R CAUDANTCNG** | 5.21 | 2.48E-07 | 1.19E-07 | | -0.34 | | 0.733 | | 0.320 | | 0.314 | | 0.289 | | 0.241 | | 0.281 | |
| **R CAUDMIDFRN** | 4.52 | 7.30E-06 | 1.03E-06 | | 2.14 | | 0.033 | | 0.049 | | 0.100 | | 0.070 | | 0.039 | | 0.037 | |
| **R CUNEUS** | -0.71 | 0.4755309 | 0.0242707 | | 1.75 | | 0.080 | | 0.095 | | 0.106 | | 0.398 | | 0.079 | | 0.073 | |
| **R ENTORHINAL** | 1.15 | 0.2501531 | 0.0134869 | | -0.45 | | 0.652 | | 0.298 | | 0.304 | | 0.481 | | 0.232 | | 0.256 | |
| **R FUSIFORM** | 2.47 | 0.013826 | 0.0008536 | | 3.29 | | 0.001 | | 0.007 | | 0.016 | | 0.038 | | 0.007 | | 0.005 | |
| **R INFPRTL** | 3.81 | 0.0001513 | 1.32E-05 | | 2.60 | | 0.010 | | 0.021 | | 0.034 | | 0.049 | | 0.017 | | 0.017 | |
| **R INFTMP** | 2.69 | 0.0074136 | 0.0004652 | | 4.14 | | 0.000 | | 0.001 | | 0.005 | | 0.007 | | 0.001 | | 0.000 | |
| **R ISTHMUSCNG** | 4.79 | 2.00E-06 | 4.49E-07 | | -0.82 | | 0.411 | | 0.248 | | 0.208 | | 0.416 | | 0.184 | | 0.212 | |
| **R LATOCC** | 1.10 | 0.2705508 | 0.014384 | | 2.32 | | 0.020 | | 0.034 | | 0.035 | | 0.057 | | 0.025 | | 0.028 | |
| **R LATORBFRN** | 4.45 | 9.81E-06 | 1.30E-06 | | 0.59 | | 0.555 | | 0.276 | | 0.310 | | 0.569 | | 0.226 | | 0.228 | |
| **R LINGUAL** | 2.10 | 0.0358558 | 0.0021786 | | 0.20 | | 0.840 | | 0.331 | | 0.306 | | 0.588 | | 0.264 | | 0.277 | |
| **R MEDORBFRN** | 4.53 | 6.86E-06 | 1.05E-06 | | -0.27 | | 0.786 | | 0.318 | | 0.301 | | 0.568 | | 0.249 | | 0.284 | |
| **R MIDTMP** | 3.20 | 0.0014219 | 9.90E-05 | | 3.79 | | 0.000 | | 0.002 | | 0.007 | | 0.005 | | 0.002 | | 0.001 | |
| **R PARAHPCMPL** | 3.23 | 0.0013144 | 9.32E-05 | | 0.05 | | 0.960 | | 0.358 | | 0.322 | | 0.583 | | 0.262 | | 0.309 | |
| **R PARACNTRL** | 2.75 | 0.0061159 | 0.0003968 | | 2.94 | | 0.003 | | 0.016 | | 0.040 | | 0.054 | | 0.012 | | 0.011 | |
| **R PARSOPRCLRS** | 4.68 | 3.36E-06 | 6.12E-07 | | 1.55 | | 0.122 | | 0.119 | | 0.196 | | 0.186 | | 0.117 | | 0.084 | |
| **R PARSORBLS** | 3.71 | 0.0002223 | 1.85E-05 | | 1.22 | | 0.222 | | 0.166 | | 0.214 | | 0.453 | | 0.148 | | 0.143 | |
| **R PARSTRNGLRS** | 3.95 | 8.66E-05 | 8.29E-06 | | 2.52 | | 0.012 | | 0.024 | | 0.075 | | 0.051 | | 0.021 | | 0.019 | |
| **R PERICLCRN** | 1.46 | 0.1437846 | 0.0079768 | | 2.02 | | 0.044 | | 0.059 | | 0.161 | | 0.279 | | 0.046 | | 0.048 | |
| **R POSTCNTRL** | 3.26 | 0.0011742 | 8.48E-05 | | 2.46 | | 0.014 | | 0.025 | | 0.037 | | 0.051 | | 0.021 | | 0.016 | |
| **R POSTCNG** | 4.52 | 7.15E-06 | 1.05E-06 | | 0.81 | | 0.420 | | 0.243 | | 0.318 | | 0.576 | | 0.192 | | 0.214 | |
| **R PRECNTRL** | 3.83 | 0.0001392 | 1.24E-05 | | 2.48 | | 0.013 | | 0.025 | | 0.052 | | 0.054 | | 0.019 | | 0.016 | |
| **R PRECUNEUS** | 4.73 | 2.74E-06 | 5.51E-07 | | 0.89 | | 0.374 | | 0.241 | | 0.318 | | 0.440 | | 0.188 | | 0.198 | |
| **R ROSANTCNG** | 4.92 | 1.07E-06 | 3.72E-07 | | -0.27 | | 0.787 | | 0.314 | | 0.310 | | 0.403 | | 0.252 | | 0.279 | |
| **R ROSMIDFRN** | 4.64 | 4.19E-06 | 7.30E-07 | | 1.70 | | 0.090 | | 0.098 | | 0.209 | | 0.248 | | 0.082 | | 0.087 | |
| **R SUPERFRN** | 5.27 | 1.78E-07 | 1.14E-07 | | 1.24 | | 0.217 | | 0.176 | | 0.236 | | 0.353 | | 0.133 | | 0.144 | |
| **R SUPERPRTL** | 4.11 | 4.40E-05 | 4.68E-06 | | 1.49 | | 0.136 | | 0.125 | | 0.161 | | 0.248 | | 0.096 | | 0.084 | |
| **R SUPERTMP** | 3.96 | 8.14E-05 | 7.99E-06 | | 2.94 | | 0.003 | | 0.014 | | 0.030 | | 0.049 | | 0.012 | | 0.010 | |
| **R SUPRAMRGNL** | 4.35 | 1.53E-05 | 1.95E-06 | | 1.81 | | 0.071 | | 0.092 | | 0.155 | | 0.110 | | 0.078 | | 0.060 | |
| **R FRNPOLE** | 3.62 | 0.0003125 | 2.55E-05 | | 1.03 | | 0.304 | | 0.205 | | 0.229 | | 0.361 | | 0.170 | | 0.185 | |
| **R TMPPOLE** | 1.97 | 0.0488773 | 0.0028784 | | 0.48 | | 0.629 | | 0.297 | | 0.319 | | 0.580 | | 0.224 | | 0.267 | |
| **R TRANSTMP** | 2.82 | 0.0049381 | 0.0003316 | | 1.20 | | 0.229 | | 0.166 | | 0.214 | | 0.446 | | 0.136 | | 0.145 | |
| **R INSULA** | 4.57 | 5.67E-06 | 9.05E-07 | | -0.37 | | 0.715 | | 0.316 | | 0.316 | | 0.547 | | 0.224 | | 0.284 | |
| **ACCUMBENS** | 4.50 | 7.81E-06 | 1.07E-06 | | -1.23 | | 0.220 | | 0.168 | | 0.210 | | 0.273 | | 0.139 | | 0.142 | |
| **AMYGDALA** | 2.09 | 0.0372292 | 0.0022267 | | 0.30 | | 0.768 | | 0.320 | | 0.250 | | 0.584 | | 0.266 | | 0.272 | |
| **BRAINSTEM** | -0.07 | 0.9473249 | 0.0477145 | | -0.66 | | 0.509 | | 0.257 | | 0.260 | | 0.354 | | 0.212 | | 0.215 | |
| **CAUD** | 4.98 | 7.90E-07 | 3.02E-07 | | -0.93 | | 0.355 | | 0.234 | | 0.218 | | 0.292 | | 0.177 | | 0.195 | |
| **HIPPOCAMPUS** | -0.76 | 0.4497134 | 0.0235818 | | -0.50 | | 0.617 | | 0.296 | | 0.294 | | 0.255 | | 0.228 | | 0.221 | |
| **PALLIDUM** | 1.66 | 0.0981532 | 0.0056078 | | -1.03 | | 0.301 | | 0.208 | | 0.212 | | 0.492 | | 0.160 | | 0.173 | |
| **PUTAMEN** | 3.73 | 0.0002056 | 1.75E-05 | | 0.71 | | 0.475 | | 0.259 | | 0.300 | | 0.588 | | 0.205 | | 0.224 | |
| **THALAMUS PRPR** | 1.58 | 0.1136406 | 0.0063972 | | 0.08 | | 0.936 | | 0.354 | | 0.348 | | 0.594 | | 0.290 | | 0.315 | |
| **VENTRALDC** | 0.06 | 0.9558229 | 0.0475173 | | -0.69 | | 0.488 | | 0.261 | | 0.257 | | 0.091 | | 0.204 | | 0.219 | |

Supplementary Table 3. **Functional connectivities showing significant differences in connectivity between functional components in progressors and non-progressors.** T-statistics and P-values before and after correction using R Studio’s *permlmer* function. Degrees of freedom df = 236. All connections between independent components with a significant permutation-corrected p<0.05 are displayed. In the main text, we avoid over-interpreting the connectivity of independent components that include primary visual cortex (IC-8, IC-19) as these regions showed worse registration accuracy. For reanalysis 5, we included Education as a covariate (R5 Education). In a separate set of models, we included baseline amyloid levels (after regressing out tracer from the mean cortical amyloid levels) as a covariate (R5 Amyloid).

| **Connection** | **T-statistic** | **P-value** | **P_PERM_** | **P_PERM_ R5 Education** | **P_PERM_ R5 Amyloid** |
| --- | --- | --- | --- | --- | --- |
| IC2-IC15 | -2.633 | 0.009 | 0.029 | 0.038 | 0.006 |
| IC4-IC15 | -2.184 | 0.029 | 0.021 | 0.049 | 0.013 |
| IC10-IC15 | -2.305 | 0.022 | 0.047 | 0.061 | 0.014 |
| IC15-IC16 | -2.448 | 0.015 | 0.045 | 0.036 | 0.022 |
| IC8-IC20 | 2.209 | 0.028 | 0.014 | 0.011 | 0.097 |

Supplementary Table 4. **Functional connectivities showing significantly different rates of change in functional connectivity between progressors and non-progressors.** T-statistics and P-values before and after correction using R Studio’s *permlmer* function. Degrees of freedom = 236. All connections between independent components with a significant permutation-corrected p<0.05 are displayed. In the main text, we avoid overanalyzing connectivity of independent components that include primary visual cortex (IC-8, IC-19) as these regions showed worse registration accuracy. For reanalysis 5, we included Education as a covariate ((R5 Education). In a separate set of models, we included baseline amyloid levels (after regressing out tracer from the mean cortical amyloid levels) as a covariate (R5 Amyloid).

| **Connectivity** | **T-statistic** | **P-value** | **P_PERM_** | **P_PERM_ R5 Education** | **P_PERM_ R5 Amyloid** |
| --- | --- | --- | --- | --- | --- |
| IC2-IC5 | 2.000 | 0.046 | 0.050 | 0.053 | 0.048 |
| IC2-IC6 | 2.360 | 0.019 | 0.025 | 0.027 | 0.019 |
| IC2-IC10 | 2.640 | 0.008 | 0.013 | 0.009 | 0.012 |
| IC8-IC10 | 3.015 | 0.003 | 0.007 | 0.008 | 0.016 |
| IC2-IC11 | 2.238 | 0.026 | 0.027 | 0.049 | 0.026 |
| IC3-IC11 | 2.385 | 0.017 | 0.023 | 0.029 | 0.022 |
| IC5-IC11 | 2.604 | 0.009 | 0.014 | 0.009 | 0.017 |
| IC8-IC12 | 2.247 | 0.025 | 0.028 | 0.033 | 0.031 |
| IC8-IC15 | 2.019 | 0.044 | 0.049 | 0.047 | 0.056 |
| IC6-IC16 | 2.042 | 0.042 | 0.050 | 0.053 | 0.053 |
| IC9-IC16 | -2.244 | 0.025 | 0.034 | 0.041 | 0.037 |
| IC6-IC18 | 2.281 | 0.023 | 0.026 | 0.029 | 0.019 |
| IC13-IC18 | 2.262 | 0.024 | 0.044 | 0.038 | 0.035 |
| IC1-IC19 | 3.378 | 0.001 | 0.008 | 0.007 | 0.005 |
| IC2-IC19 | 3.184 | 0.002 | 0.010 | 0.014 | 0.012 |
| IC6-IC19 | 2.776 | 0.006 | 0.021 | 0.008 | 0.014 |
| IC7-IC19 | 3.347 | 0.001 | 0.025 | 0.034 | 0.02 |
| IC8-IC19 | 2.160 | 0.031 | 0.038 | 0.042 | 0.031 |
| IC10-IC19 | 2.253 | 0.025 | 0.029 | 0.035 | 0.033 |
| IC6-IC21 | 2.372 | 0.018 | 0.017 | 0.015 | 0.012 |
| IC13-IC21 | 2.620 | 0.009 | 0.018 | 0.016 | 0.019 |

Supplementary Table 5. **Correlation statistics between amyloid PCA1 and significant functional connectivities.** As described in the methods section of the main text, we computed slopes of amyloid accumulation and change in brain network functional connectivity as annual rates of change. We then computed a principal component analysis on the amyloid slopes of the regions with significant progression group by time interactions and used the first principal component (PCA1) in correlation analyses with each of the independent component pairs that also showed significant progression group by time interactions. Participants with amyloid slopes with absolute values greater than 1 were excluded, as were those with functional connectivity slopes with absolute values greater than 2. Both uncorrected and multiple comparison-corrected p-values are shown.

| **Connectivity** | **Pearson’s R** | **Degrees of freedom** | **P-value** | **P_FDR_** |
| --- | --- | --- | --- | --- |
| IC2-IC5 | 0.1955 | 221 | 0.0034 | 0.003966667 |
| IC2-IC6 | 0.2135 | 221 | 0.0013 | 0.001605882 |
| IC2-IC10 | 0.2194 | 221 | 9.70E-04 | 0.001358671 |
| IC8-IC10 | 0.2703 | 221 | 4.31E-05 | 0.000134603 |
| IC2-IC11 | 0.2621 | 221 | 7.43E-05 | 0.000173254 |
| IC3-IC11 | 0.2922 | 221 | 9.15E-06 | 4.81E-05 |
| IC5-IC11 | 0.2627 | 221 | 7.15E-05 | 0.000173254 |
| IC8-IC12 | 0.2567 | 221 | 1.06E-04 | 0.000199884 |
| IC8-IC15 | 0.2935 | 221 | 8.35E-06 | 4.81E-05 |
| IC6-IC16 | 0.2147 | 221 | 1.30E-03 | 0.001605882 |
| IC9-IC16 | 0.0463 | 221 | 0.4914 | 0.4914 |
| IC6-IC18 | 0.2271 | 221 | 6.34E-04 | 0.001023906 |
| IC13-IC18 | 0.2555 | 221 | 1.14E-04 | 0.000199884 |
| IC1-IC19 | 0.1864 | 221 | 0.0052 | 0.005747368 |
| IC2-IC19 | 0.3181 | 221 | 1.24E-06 | 1.30E-05 |
| IC6-IC19 | 0.2195 | 221 | 9.65E-04 | 0.001358671 |
| IC7-IC19 | 0.3441 | 221 | 1.35E-07 | 2.83E-06 |
| IC8-IC19 | 0.2764 | 221 | 2.85E-05 | 0.00011954 |
| IC10-IC19 | 0.1017 | 221 | 0.1301 | 0.136605 |
| IC6-IC21 | 0.2697 | 221 | 4.49E-05 | 0.000134603 |
| IC13-IC21 | 0.256 | 221 | 1.11E-04 | 0.000199884 |

Supplementary Figure 2. Relative Distribution of Amyloid Levels in Progressors vs Non-Progressors. Violin plots of a) mean cortical amyloid levels at baseline, b) mean subcortical amyloid levels at baseline, c) mean cortical amyloid levels at last follow-up, and d) mean subcortical amyloid levels at last follow-up. Raw values are shown.


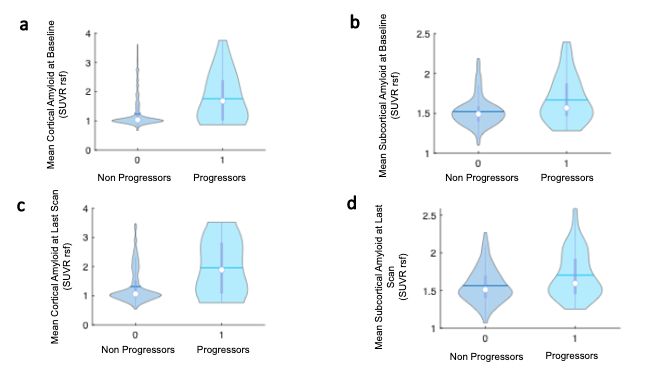


Supplementary Figure 3. Relative Distribution of Amyloid Levels in Progressors vs Non-Progressors. Violin plots of a) mean cortical amyloid levels at baseline, b) mean subcortical amyloid levels at baseline, c) mean cortical amyloid levels at last follow-up, and d) mean subcortical amyloid levels at last follow-up. Model fitted values are shown.


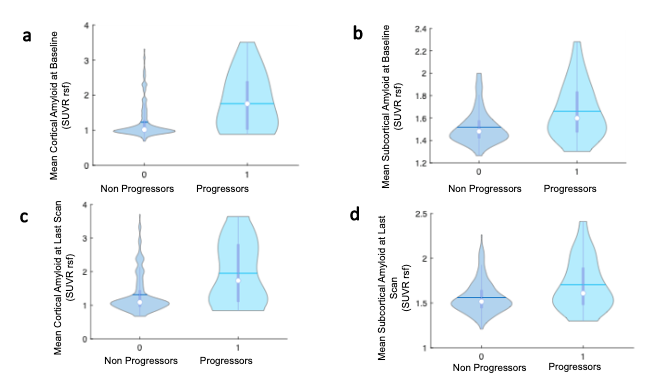


**Spatial relationship between longitudinal amyloid and FC results**

We found that progressors had faster accumulation of amyloid in lateral and inferior temporal, motor (postcentral gyrus), and inferior frontal regions compared to non-progressors. Therefore, we sought to quantify the overlap between these regions with the spatial maps of networks that showed increased connectivity over time in progressors vs. non-progressors. We first binarized the network maps (*mri_binarize*) with a threshold of 3, which was used to extract the timeseries. We then mapped each binarized network map onto the Desikan-Killiany parcellation that was used in the amyloid-β analyses by calculating the proportion of each Desikan-Killiany region occupied by the respective network (*mri_segstats*). The mappings between the Desikan-Killiany parcellation and each network maps are available at <https://github.com/gemmamoffat/OASIS3_LMEanalyses>. We then identified ICs with spatial maps that were at least partially located in the regions that showed a significant group x time interaction on amyloid levels.

For instance, IC-11 occupied 64.96% of the right postcentral and 29.64% of the right precentral regions, both of which showed a significant interaction between progressor group and time for amyloid levels. Importantly, this network was primarily localized in these motor regions, as the only other regions occupied by IC-11 were insula (27.78% of insula and 11.69% of caudal anterior cingulate occupied by IC-11). IC-1 on the other hand occupied a variety of regions, e.g. taking up 25.0% of the middle temporal region and 16.55% of the inferior parietal region which showed a significant interaction. At the same time, however, the IC-1 map was also located in regions that did not show a significant interaction for amyloid levels such as medial orbitofrontal (37.24%) or frontal pole (51.49%). Notably, IC-13 also covered many of the regions that showed a significant interaction for amyloid levels, including pars triangularis (32.73%), superior temporal (28.16%), inferior temporal (22.65%) and inferior parietal (12.04%). IC-5 covered a mix of regions, many of which showed a significant interaction in amyloid analyses (inferior parietal, 50.93%, inferior temporal 31.11%, middle temporal 47.66%). However, IC-5 also covered regions with no significant interactions (caudal middle frontal, 73.21%, rostral middle frontal 59.60%, superior frontal 33.73%, superior parietal 30.73%, supramarginal 32.54%). IC-3 also covered a number of regions showing an interaction in amyloid analyses including the inferior temporal inferior parietal, precentral, postcentral, middle temporal, and pars triangularis regions. IC-2 also showed a substantial overlap with regions with a significant interaction in amyloid analyses (including fusiform, inferior parietal, inferior temporal, and precentral regions). On the other hand, brain maps for IC-6, IC-8, IC-16, IC-19 and IC-21 showed a lot less overlap with regions showing a group x time interaction on amyloid levels. More details on the overlap of spatial maps can be found in Supplementary Figure 4.


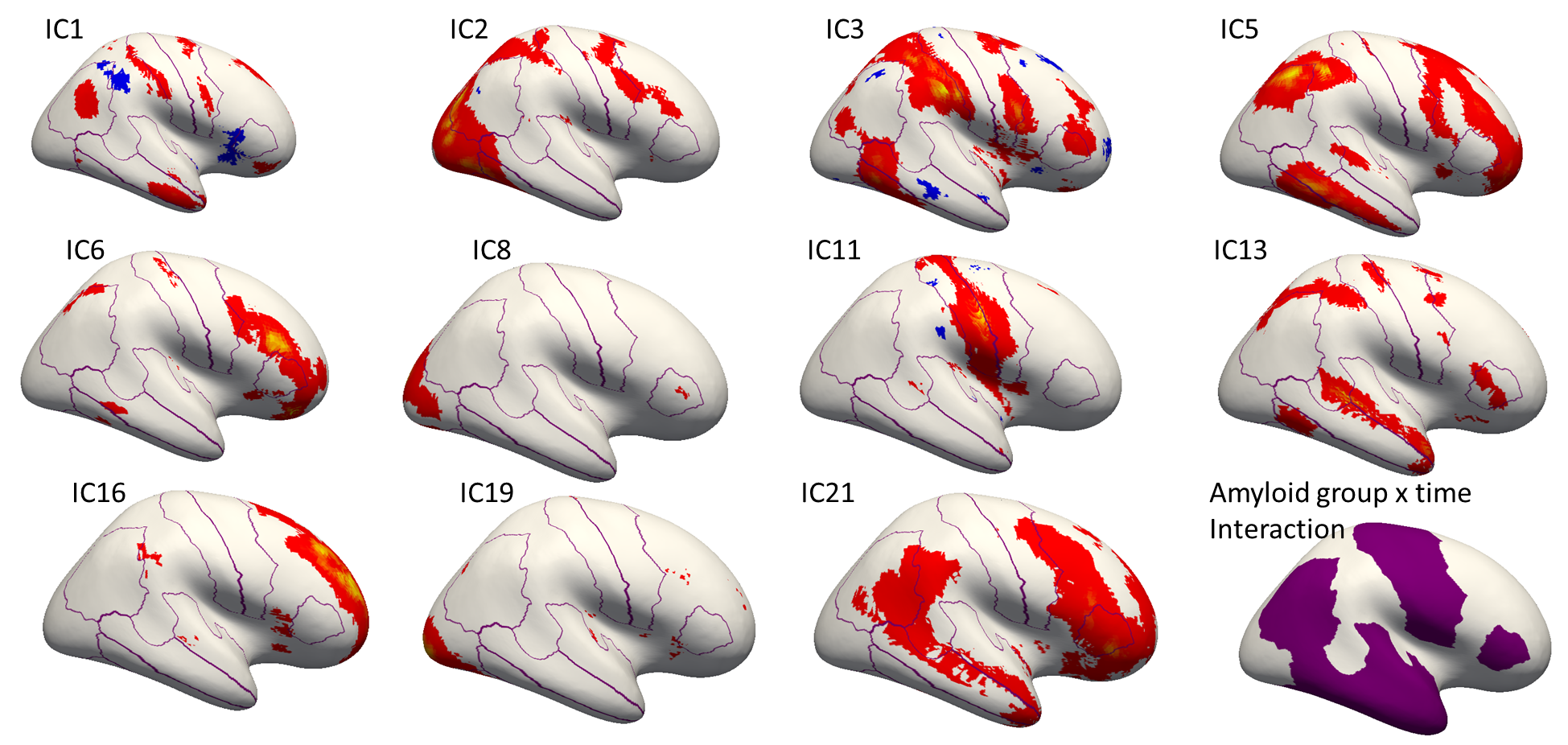


Supplementary Figure 4. Overview of spatial overlap between functional connectivity networks (Independent Components, IC) and Desikan-Killiany regions showing a significant group x time interaction in functional connectivity and amyloid analyses, respectively. Desikan-Killiany regions showing a significant interaction in amyloid analyses are highlighted with a purple outline and are shown in purple in the bottom right panel. Thresholded brain maps for ICs (Z>3) are shown in red. Briefly, most overlap was seen for IC-11, IC-2, IC-3, IC-5 and IC-13. Frontoparietal networks such as IC-6, IC-21, IC-16 and visual networks such as IC-8 and IC-19 showed less overlap.
